# A nematode demographics assay in transgenic roots reveals no significant impacts of the *Rhg1 *locus LRR-Kinase on soybean cyst nematode resistance

**DOI:** 10.1186/1471-2229-10-104

**Published:** 2010-06-09

**Authors:** Sara Melito, Adam L Heuberger, David Cook, Brian W Diers, Ann E MacGuidwin, Andrew F Bent

**Affiliations:** 1 Department of Plant Pathology, University of Wisconsin - Madison, Madison, WI 53706 USA; 2 Department of Crop Sciences, University of Illinois at Urbana-Champaign, Urbana, IL 61801 USA

## Abstract

**Background:**

Soybean cyst nematode (*Heterodera glycines*, SCN) is the most economically damaging pathogen of soybean (*Glycine max*) in the U.S. The *Rhg1 *locus is repeatedly observed as the quantitative trait locus with the greatest impact on SCN resistance. The Glyma18g02680.1 gene at the *Rhg1 *locus that encodes an apparent leucine-rich repeat transmembrane receptor-kinase (LRR-kinase) has been proposed to be the SCN resistance gene, but its function has not been confirmed. Generation of fertile transgenic soybean lines is difficult but methods have been published that test SCN resistance in transgenic roots generated with *Agrobacterium rhizogenes*.

**Results:**

We report use of artificial microRNA (amiRNA) for gene silencing in soybean, refinements to transgenic root SCN resistance assays, and functional tests of the *Rhg1 *locus LRR-kinase gene. A nematode demographics assay monitored infecting nematode populations for their progress through developmental stages two weeks after inoculation, as a metric for SCN resistance. Significant differences were observed between resistant and susceptible control genotypes. Introduction of the *Rhg1 *locus LRR-kinase gene (genomic promoter/coding region/terminator; Peking/PI 437654-derived SCN-resistant source), into *rhg1*^- ^SCN-susceptible plant lines carrying the resistant-source *Rhg4*^*+ *^locus, provided no significant increases in SCN resistance. Use of amiRNA to reduce expression of the LRR-kinase gene from the *Rhg1 *locus of Fayette (PI 88788 source of *Rhg1*) also did not detectably alter resistance to SCN. However, silencing of the LRR-kinase gene did have impacts on root development.

**Conclusion:**

The nematode demographics assay can expedite testing of transgenic roots for SCN resistance. amiRNAs and the pSM103 vector that drives interchangeable amiRNA constructs through a soybean polyubiqutin promoter (Gmubi), with an intron-GFP marker for detection of transgenic roots, may have widespread use in legume biology. Studies in which expression of the *Rhg1 *locus LRR-kinase gene from different resistance sources was either reduced or complemented did not reveal significant impacts on SCN resistance.

## Background

Soybean cyst nematode (SCN, *Heterodera glycines*) is an obligate, sedentary endoparasite that is consistently the most damaging pest of soybean in the U.S. [1]. Once SCN is present in a field it cannot feasibly be eradicated.

The SCN life cycle consists of five stages. After the first molt within the egg, SCN second stage juveniles (J2) hatch, move through the soil, penetrate roots and move toward the vascular cylinder [2,3]. Migratory juveniles select a host cell in the cortex, endodermis, or pericycle and induce host cell fusion as part of the formation of a permanent feeding site called a syncytium. At this point the nematode becomes sedentary and differentiates to the third (J3) and fourth (J4) juvenile stages and then matures to an adult female or male. Males undergo a metamorphosis to resume a vermiform shape at the J4 stage and migrate back out of the root to fertilize adult females. Following fertilization, the female produces eggs, most of which remain inside the body. After dying, the female body develops into a hardened cyst that encases the eggs. At 25°C, some nematodes reach the adult stage 12 days after entering roots, and most become adults by 30 days post-infection [4].

Soybean cyst nematodes infect and grow in the roots of both resistant and susceptible cultivars [2,5]. Nematode growth and development depends on the successful establishment and maintenance of a syncytium, and impairment of the syncytium can give outcomes that range from reduced growth and reproduction to death. The available SCN resistance in soybean is partial, and can be observed as a reduced number of females that develop compared to the number that develop on similarly inoculated susceptible cultivar controls [6]. In intact soybean plants, resistance is often expressed as the female index (FI): the number of fully developed females (cysts) on the tested soybean genotype divided by the number of females on a susceptible standard [2,7]. Soybean cultivars are generally classified as strongly resistant to SCN if the FI is less than 10%; partial levels of resistance can also be useful [8].

The SCN resistance in current commercially grown soybeans is derived from a very small number of sources [9]. These sources include 'Peking' (PI 548402), PI 88788 and PI 437654, which have each been shown to carry resistance loci effective against multiple nematode races [10,11]. Inheritance of resistance to SCN was first reported in the 'Peking' plant introduction, and three genes for resistance (*rhg1*-*rhg3*) were assigned and initially classified as recessive [12]. Of the resistance sources, PI 88788 has been the most widely used in breeding programs. More than 95% of the SCN resistant cultivars available for planting in Illinois during 2009 received their resistance from this PI [13].

The *Rhg1 *locus has been shown to have the greatest impact on SCN development in several resistance sources including Peking, PI 88788, PI 437654, PI 209332 and PI 90763. This locus provides resistance to many common SCN populations such as Hg type 0 (race 3) [10]. Multiple research groups have mapped the *Rhg1 *locus to a sub-telomeric region on chromosome 18 [11,14,15], to a location approximately 0.4 centimorgans (cM) from the simple sequence repeat (SSR) marker Satt309 [16] (chromosome 18 was formerly known as linkage group G; http://www.phytozome.net/[17]). Although originally reported as a recessive locus, "*rhg1" *has more recently been characterized as exhibiting incomplete dominance. Soybean lines heterozygous for resistant and susceptible alleles at the *Rhg1 *locus often allow SCN cyst formation at a rate intermediate between that of plants genotyped with Satt309 as homozygous resistant or homozygous susceptible at the *Rhg1 *locus [18-20](Kim et al. submitted; incomplete dominance also has been observed in unpublished work with the Ina × E98076 material used in this study). A second QTL (*Rhg4*) has been identified as being necessary for full resistance to some SCN populations in Peking and in PI 437654, but not PI 88788 or PI 209332 [7,9]. *Rhg4 *exhibits dominant gene action, and in Peking-derived material the relevant alleles of both *Rhg1 *and *Rhg4 *are necessary to exhibit the full resistance phenotype. Other loci that make smaller and/or more race-specific contributions to SCN resistance have also been identified throughout the soybean genome, but often a given locus was identified in only one study [10,21-25].

Cytological studies suggest that Peking-type resistance displays host cell necrosis and cell wall appositions not seen in PI 88788 type resistances in response to SCN Hg type 0 [26,27]. The Peking and PI 88788 *Rhg1 *sources also exhibit distinct differential behaviors in their strength of resistance against particular SCN test populations, suggesting at least partially different mechanisms in the SCN resistance controlled by different *Rhg1 *alleles [28,29].

Two groups first filed applications with the U.S. Patent Office in 2000 identifying apparent leucine-rich repeat transmembrane receptor-kinase (LRR-kinase) genes currently annotated as Glyma18g02680.1 and Glyma08g11350.1 http://www.phytozome.net/[17] as the likely SCN resistance genes at both *rhg1 *and *Rhg4 *[18,30], see also [19,31]. Sequences supporting the claims were released to Genbank between 2000 and 2005. The basis for these claims was the presence of these genes at the *Rhg1 *and *Rhg4 *loci, their similarity to the known rice bacterial blight resistance gene *Xa21*, and the presence of derived amino acid sequence differences between the alleles from resistant and susceptible plant genotypes. However, a decade later, no functional evidence for a role of these LRR-kinase genes in SCN resistance has been reported in a peer-reviewed forum. There are numerous reasons why identification of the *Rhg1 *gene that confers SCN resistance is a high priority, including the extreme economic significance of SCN for yield loss, the major reliance on the *Rhg1 *locus in commercial soybean breeding, the detection of SCN populations that overcome currently available *Rhg1*-mediated resistance, the potential to respond to these challenges with engineered improvements in *Rhg1*, and basic scientific interest in the nature of plant resistance to SCN.

An experimentally tractable transgenic assay system is a crucial component for the identification, study and manipulation of genes controlling SCN resistance as well as many other soybean traits. Generation of transgenic fertile soybean lines is still a difficult and expensive process that requires close to a year to obtain transgenic seed lines. *Agrobacterium rhizogenes *has been used by many researchers as a transgene delivery system to study legume root biology, both in soybean and *Medicago truncatula *[32-36]. Transgenic roots generated using *A. rhizogenes *retain the SCN resistance phenotypes of the parental soybean genotypes, and have been used to test genes that may impart resistance [37,38].

Function is often attributed to specific genes by the methods of gene mutation/positional cloning, phenotypic complementation via transformation with a cloned full-length gene, and/or by gene silencing. Experimental silencing of plant genes has generally been elicited using hairpin RNA-forming inverted repeat DNA constructs or by virus-induced gene silencing [39-42]. All silencing approaches are prone to incomplete penetrance (partial silencing, often for unpredictable reasons), but have nevertheless been useful for assigning function to specific genes, and for engineering useful traits. These gene silencing techniques have been used effectively in soybean and other legumes [43-49]. With the more recent discovery of endogenous microRNAs as a major mode of gene regulation in many eukaryotes, artificial microRNA (amiRNA) methods have been developed for investigator-initiated silencing of target genes [50-52]. Potential advantages of amiRNAs may include better penetrance, absence of undesired phenotypes associated with VIGS virus infections, and the capacity to limit off-target silencing of related sequences by elicitation with constructs specific for very short (19-24 bp) target sequences. However, use of amiRNA technology with soybean, *M. truncatula*, *Phaseolus *or other legumes has not been reported.

For the present study we refined assays that test SCN resistance in transgenic roots generated with *A. rhizogenes*. A nematode demographic assay was developed that discriminates resistant and susceptible responses by monitoring the infecting population for progression through nematode life stages. We also developed an amiRNA vector system for induction of gene silencing in legume roots using *A. rhizogenes *assay systems. We used these tools to investigate the impact on SCN resistance of the LRR-kinase gene from the *Rhg1 *genomic region (the candidate SCN resistance gene). Our experiments expressing the LRR-kinase from a resistant (Peking/PI 437654-source) *Rhg1 *locus in susceptible test lines, with or without the resistant allele at *Rhg4*, and silencing the LRR-kinase (PI 88788 source) in resistant lines, provided no evidence for a contribution of this gene to SCN resistance.

## Results

### Scoring resistance using a hairy root/nematode demographics assay

**Figure 1:**
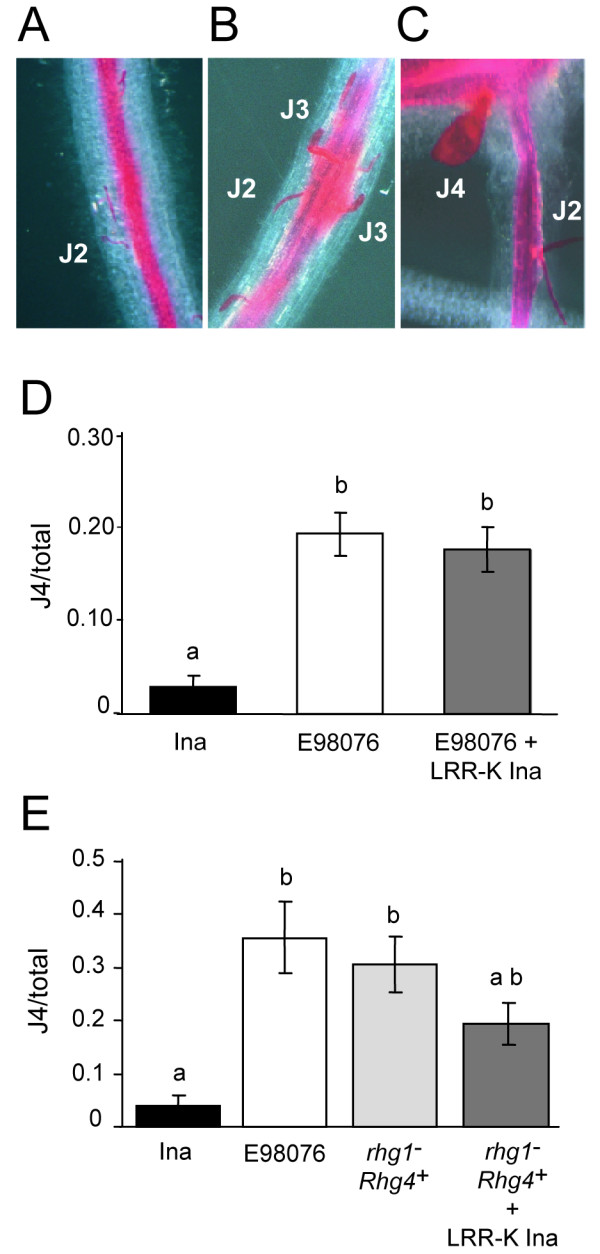
**Nematode demographics assay for SCN resistance in roots transformed with a full-length *Rhg1*-locus LRR-Kinase gene**. **A, B, C**, Representative examples of SCN developmental stages on transgenic soybean hairy roots 15 days after infection; cleared root sections were stained with acid fuchsin, which stains nematodes as well as root vascular bundles. **A**, Early parasitic second-stage juveniles (J2). **B**, Late parasitic J2s and early J3s. **C**, J4 or adult female. **D**, Ratio of total infecting nematode population that developed to J4 or adult female stage two weeks after inoculation onto *A. rhizogenens*-transformed roots of Ina, E98076 (both transformed with empty vector), or E98076 + LRR-K Ina (transformed with the Glyma18g02680.1 *Rhg1*-locus LRR-Kinase promoter/gene/terminator). Mean ± std error of mean; data combined from two independent experiments; bars with the same letter above are not significantly different (ANOVA, Tukey pairwise comparison, p < 0.001). **E**, Similar to D, except that "*rhg1*^- ^*Rhg4*^*+ *^are putative *rhg1*^-^**/***rhg1*^-^; *Rhg4*^*+*^**/***Rhg4*^*+ *^progeny lines from Ina × E98076 that were genotyped as carrying the E98076 (susceptible) allele of a marker linked to *Rhg1 *and the Ina (resistant) allele of a marker linked to *Rhg4*. All the lines were transformed with empty vector except "*rhg1*^- ^*Rhg4*^*+ *^+ LRR-K Ina" was transformed with *Rhg1*-locus LRR-kinase gene (including native promoter and terminator). Mean ± std error of mean are from two independent experiments; bars with the same letter above are not significantly different (ANOVA, Tukey pairwise comparison, p < 0.05).

In soybean, *Medicago truncatula *and other plants for which stable transformation is difficult, transgenic "hairy roots" generated through *A. rhizogenes*-mediated transformation are often used to investigate gene function. A number of groups have previously shown that the resistance response to SCN in *A. rhizogenes*-derived roots correlates well with the SCN resistance phenotypes of intact soybean plants [37,38]. Cyst formation is typically counted after 4 weeks but because the root tissues used often exhibit significantly reduced vigor 3-4 weeks into such assays, and to expedite and possibly improve resistance scoring, we investigated the scoring of resistance phenotypes at an earlier time point. Host resistance to SCN is often manifested as arrested development at specific life stages [38,53], so we developed a "nematode demographics assay" in which SCN-infested transgenic root sections were stained with acid fuchsin approximately 2 weeks after inoculation with nematodes. Using a stereo dissecting microscope, the number of nematodes at each growth stage (J2, J3, or J4 + adult) was recorded for each root segment (Figure 1A,B,C). Data are expressed as a ratio: the number of nematodes within a root segment with an appearance resembling the J4 + adult, or J3 + J4 + adult, growth stage divided by the total number of nematodes (J2 + J3 + J4 + adult) within the root segment. Relative SCN resistance or susceptibility is determined by comparison to the demographic data for known SCN-resistant or SCN-susceptible host genotypes tested within the same experiment. The results of two such assays are shown in Figure 1D and 1E. In these and other experiments (below and data not shown) there was a reproducible significant difference between the SCN resistance phenotypes determined for known SCN-susceptible and SCN-resistant control genotypes.

### No elevation of SCN resistance observed in susceptible genotypes transformed with the *Rhg1 *locus LRR-kinase gene from resistant germplasm

The *Rhg1 *locus of soybean has been shown in numerous studies to be the QTL with the greatest phenotypic impact on SCN resistance, and previous patents and other reports have indicated but not demonstrated that the Glyma18g02680.1 gene at the *Rhg1 *locus that encodes an apparent leucine-rich repeat transmembrane receptor kinase (LRR-K) is the source of this SCN resistance. We isolated this LRR-K gene from the *Rhg1 *locus using genomic DNA from the soybean cultivar Ina that we confirmed to exhibit SCN resistance in a standard whole plant/cyst formation assay immediately prior to its use in DNA isolation. Ina was used because of available progeny lines that are described below; Ina has Peking, PI 437654 and PI 88788 in its pedigree but carries an LRR-K at the *Rhg1 *locus that is identical in sequence to the Peking allele and also similar to the PI437654 allele. Multiple independent PCR products were isolated and sequenced, and the sequences were compared to each other and to the Peking sequence published in [30] to identify the best clone. An *Agrobacterium*-compatible binary vector pGWB1::erGFP7INT carrying a screenable intron-GFP marker was constructed to transform soybean with candidate SCN resistance genes in full-gene complementation experiments. An insert of 6.9 kb was added to pGWB1::erGFP7INT and included approximately 2 kb of genomic upstream DNA, the predicted 2757 bp LRR-K coding region + intron, and approximately 800 bp of downstream DNA. SCN-susceptible E98076 was then transformed with this construct to test the impact of this LRR-K allele on SCN development. Using the nematode demographics assay described above, roots from soybean line E98076 transformed with the *Rhg1 *locus LRR-K did not display increased resistance to SCN; nematode development progressed similarly to what was observed on E98076 susceptible control roots transformed with the empty pGWB1::erGFP7INT vector (Figure 1D). E98076 transformed with either an empty plasmid vector or with the *Rhg1 *locus LRR-K were, however, both significantly more susceptible to SCN than the resistant line Ina (p < 0.001).

The *Rhg4 *locus in Peking and PI 437654 also contributes to the full SCN resistance phenotype [10]. SCN-susceptible soybean genotypes with endogenous SCN-susceptible alleles at both *Rhg1 *and *Rhg4*, which are then transformed with a resistant allele only of *Rhg1*, might not display a resistant phenotype. To further test the impact of the *Rhg1 *locus LRR-K gene on SCN development, *rhg1*^- ^lines carrying the SCN-resistant Peking *Rhg4 *locus were tested by complementation. Microsatellite markers linked to *Rhg1 *and *Rhg4 *were used to identify Ina × E98076 progeny lines with a (-/-, +/+) genotype (*rhg1*_E98076_*/rhg1*_E98076_, *Rhg4*_Ina_/*Rhg4*_Ina_). These lines were transformed with the cloned Ina *Rhg1*-locus LRR-K gene and assayed for SCN development. In the combined results from two independent experiments, no significant change in the resistance to SCN was detected after transformation of these genotypes with the Ina (Peking)-derived *Rhg1 *locus LRR-K gene (Figure 1E). The two *rhg1*^- ^lines transformed with an empty vector were significantly more susceptible to SCN than resistant Ina (p < 0.01), while the *rhg1*_E98076_*/rhg1*_E98076_, *Rhg4*_Ina_/*Rhg4*_Ina _genotype transformed with the *Rhg1 *locus LRR-K gene was not significantly more resistant than the same genotype transformed with empty vector. The mean resistance of roots transformed with the Ina LRR-K gene may have been slightly greater but not to a significant extent, in contrast to the statistically significant difference between the Ina and E98076 control lines. When the nematode demographics data for these same experiments were expressed as [(J3+J4)/total], the results for *rhg1*_E98076_*/rhg1*_E98076_, *Rhg4*_Ina_/*Rhg4*_Ina _genotypes transformed with empty vector or vector + *Rhg1 *locus LRR-K gene were even more similar (mean ± std error 0.59 ± 0.09 for empty vector and 0.49 ± 0.09 for LRR-K Ina; P = 0.85 for ANOVA Tukey simultaneous tests).

### Construction of an amiRNA gene silencing vector

**Figure 2:**
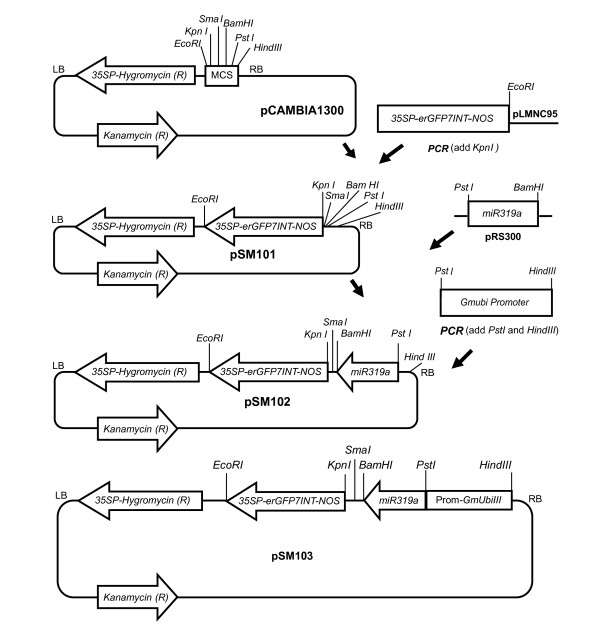
**Construction of pSM103, a plasmid for amiRNA-mediate gene silencing in legume roots**. pSM101 is an intron-GFP7 *Agrobacterium*-compatible binary plasmid for plant transformation, derived from pCAMBIA1300 by addition between T-DNA borders of a 35S promoter-erGFP7INT-NOS terminator (Mankin et al. 2001). pSM102 is pSM101 with a promoterless Arabidopsis miRNA *miR319a *gene. pSM103 is pSM102 with the miR319a driven by a promoter from the soybean ubiquitin-3 gene for strong expression in legume roots. After opening with PstI and BamHI to remove the miR319a, pSM103 can receive user-designed amiRNA gene silencing constructs from the widely used plasmid pRS300 (http://wmd3.weigelworld.org/; Schwab et al 2006), or other constructs.

**Figure 3:**
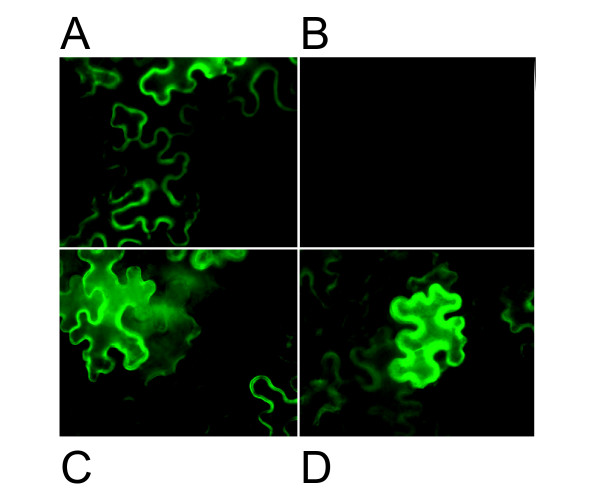
**Transient expression of pSM101 and pSM103 in *Nicotiana benthamiana *leaves**. **A, B, C**, The new binary vectors pSM101 and pSM103 were tested for GFP7 expression in *Nicotiana benthamiana *leaves. Bacterial suspensions were infiltrated through the abaxial surface of the leaf and GFP7 expression was monitored three days later by fluorescence microscopy. Leaves were infiltrated with: **A**, positive control *A. tumefaciens GV3101 *pGWB1::35S-erGFP7INT-NOS; **B**, negative control untransformed *A. tumefaciens GV3101; ***C **&**D**, *A. tumefaciens GV3101 *pSM101 (C) or pSM103 (D) that carry intron GFP7 constructs.

We sought to use gene silencing as an independent means to test for contributions of the *Rhg1 *locus LRR-kinase gene toward SCN resistance. To facilitate this work, we constructed a new vector for gene silencing in transgenic soybean roots. The binary vector pSM103 (pCAMBIA1300::35SP-erGFP7INT-NOS, Gmubi promoter, mi319RNAa) was designed to meet the following requirements: 1) efficient cloning of artificial microRNAs, 2) constitutive strong amiRNA expression in legume roots as well as other tissues [54], 3) reliable identification of transgenic roots by GFP screening using an intron-containing GFP that is not expressed in bacteria, 4) competence for transferring inserted DNA into plant genomes via *Agrobacterium*, and 5) high transformation efficiency. The steps taken to develop the vector are shown in Figure 2, as the intermediate constructs may also be of use and are available upon request. To initially confirm GFP7 expression *in planta*, pSM101 and pSM103 were infiltrated into *N. bentamiana *leaves using *A. tumefaciens GV3101*. Positive and negative controls (pGWB1::35SP-erGFP7INT-NOS, and *A. tumefaciens GV3101 *without any vector) did or did not produce green fluorescence respectively, as expected (Figure 3A and 3B). erGFP7INT expression was phenotypically detected with use of vectors pSM101 and pSM103 (Figure 3C and 3D).

### Silencing of the *Rhg1 *locus LRR-Kinase in transgenic roots

**Figure 4:**
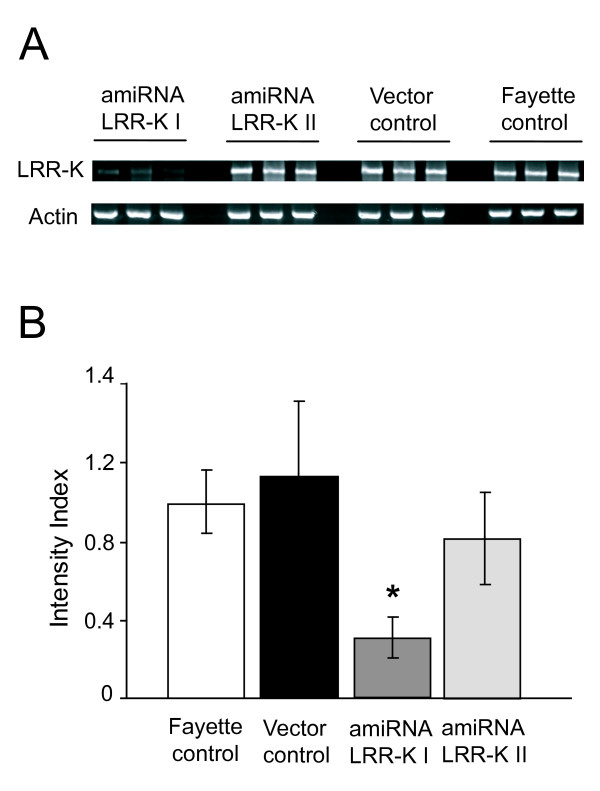
**amiRNA-mediated silencing of expression of the *Rhg1*-locus LRR-kinase gene**. **A**, Semi-quantitative RT-PCR to monitor expression level of the *Rhg1*-locus LRR-kinase gene Glyma18g02680.1/TC204550 (LRR-K) in Fayette transgenic roots generated using pSM103-derived plasmids expressing amiRNA GmLRR-K I or amiRNA GmLRR-K II, or GFP7-positive Fayette transgenic roots generated using the negative control vector pSM103 (vector control), or GFP-negative Fayette hairy roots. Actin expression was monitored in parallel from the same samples as a control. Results from three independent roots are shown for each construct; gels were stained with ethidium bromide. Similar results were observed in a second experiment using independently derived roots. **B**, Relative band intensity of semi-quantitative RT-PCR products. Data from the experiment shown in part A and from a similar but independent transformation experiment are shown. Band intensities were normalized to the mean value for the three Fayette control roots from the same experiment. Mean ± std error of mean are shown; statistical analysis was performed on the normalized data. Roots expressing amiRNA GmLRR-K I exhibited a significantly reduced mRNA abundance for the *Rhg1*-locus LRR-kinase gene, as indicated with the * (ANOVA; Tukey comparison of means; p = 0.0023).

For silencing experiments, the *Rhg1 *locus from the more widely used PI 88788 source of *Rhg1 *was tested using the soybean cultivar Fayette. Cotyledons of Fayette were transformed with *A. rhizogenes *containing pSM103 derivatives carrying one of two different amiRNAs that each were designed to specifically target the Gm18 *Rhg1 *locus LRR-K gene. To determine the effectiveness of the two amiRNAs, RNA was extracted from three independent transgenic hairy roots for each construct. Semi-quantitative RT-PCR was performed in each of two independent biological replicate experiments as shown in Figure 4A. The semi-quantitative RT-PCR was also performed using Fayette GFP-negative hairy roots to determine the basal expression level of the *Rhg1 *locus LRR-kinase gene, and using Fayette GFP-positive roots to verify that unmodified pSM103 does not interfere with expression of the *Rhg1 *locus LRR-kinase gene. No significant differences were found between the two Fayette controls (Figure 4A and 4B). ANOVA was utilized to evaluate differences in expression of the *Rhg1 *locus LRR-K gene. As shown in Figure 4A and 4B, at 35 cycles only the "amiRNA LRR-K I" construct was able to substantially reduce the expression level (mRNA abundance) of the *Rhg1 *locus LRR-kinase gene in transgenic roots (ANOVA p < 0.05). No statistical difference was found for the amiRNA LRR-K II, but a trend of partial down regulation was observed (Figure 4B). The actin control did not show a significant difference among the samples.

### Impacts on Transformation Efficiency and Root Development

**Figure 5:**
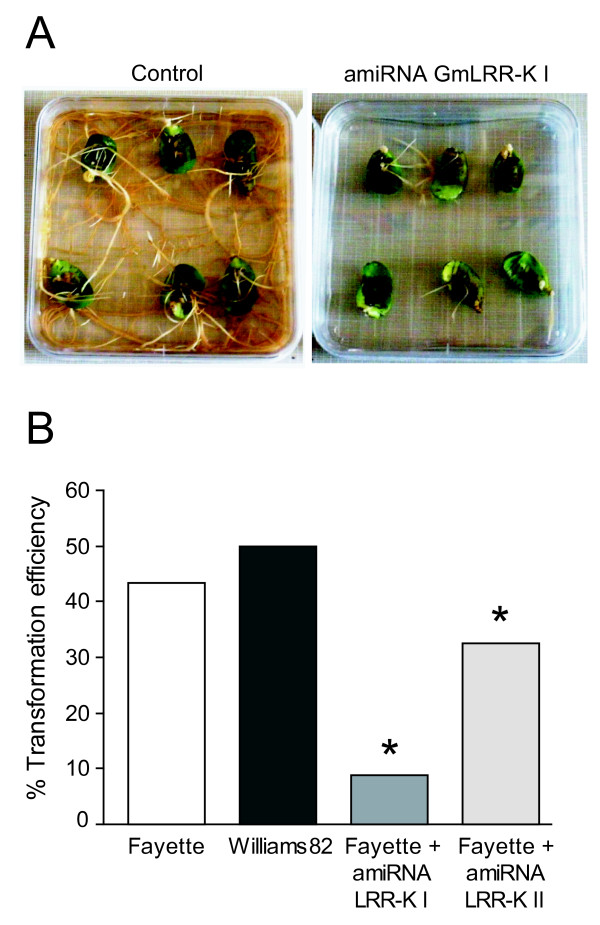
**Root production by soybean cotyledons transformed using *A. rhizogenes *carrying amiRNA silencing constructs**. **A**, Fayette cotyledons three weeks after treatment with *A. rhizogenes *pSM103 (control, plasmid carries *A. thailiana *miRNA319a lacking any known soybean target), or with *A. rhizogenes *pSM103 derivative carrying amiRNA GmLRR-K I. Representative explants are shown. **B**, Transformation efficiency reduction when *A. rhizogenes *deliver amiRNAs targeting the *Rhg1*-locus LRR-kinase gene. Efficiency is the % of treated cotyledons that produced at least one GFP7-positive root segment; * indicates significant difference from Fayette control (χ^2^-test, see text). Cotyledons transformed with pSM103 derivates carrying amiRNA GmLRR-K I developed roots approximately two weeks later than those transformed with the control pSM103 vector, and developed fewer roots per cotyledon. This effect was also present, but less dramatic in the cotyledons that received the amiRNA GmLRR-K II.

Transformation efficiency was evaluated 3 weeks after *A. rhizogenes *application, with GFP used as a visual marker for successful transformation. Transformation frequency was calculated as the number of cotyledons with at least one transgenic hairy root divided by the total number of inoculated cotyledons. Fayette and Williams 82 transformed with pSM101, pSM103 and pGWB1::35SP-erGFP7INT showed transformation efficiencies ranging from 43.33% to 50%. No significant difference in transformation efficiency was observed between the two genotypes transformed with the different control vectors (Figure 5B). However, when Fayette cotyledons were transformed with amiRNA GmLRR-K I and amiRNA GmLRR-K II, decreased transformation efficiency was observed. Cotyledons transformed with the two LRR-kinase amiRNA constructs developed hairy roots roughly two weeks later, and had lower transformation efficiencies than the controls. In particular, the amiRNA GmLRR-K I gave the lowest transformation efficiency (8.37%) and the resulting roots grew more slowly (Figure 5A and 5B). Based on two independent transformation experiments using 20 cotyledons of each genotype, a Chi-square test revealed no significant different between Williams and Fayette controls for root transformation efficiency (χ^2 ^P = 0.518), while a significant difference was observed for amiRNAGmLRR-K I (χ^2 ^P = 3.56e-10) and amiRNA GmLRR-K II (χ^2 ^P = 1.156e-4) relative to the expected rate established from Fayette controls.

To evaluate potential artifactual impacts of the amiRNA vector in plants lacking the target gene, a transformation experiment was performed using *Medicago truncatula*. The Fayette TC204550 sequence has only 81% identity with *M. truncatula *Chromosome 5 clone mth2-155g22 (CU302335). *M. truncatula *seeds were transformed with *A. rhizogenes *to deliver the same silencing constructs tested in soybean. Three plates, each with 12 germinated plants, were used for each construct. Three weeks after the treatment, no difference in root development was observed between *M. trucatula *seedlings transformed with the two amiRNA GmLRR-K constructs in comparison to the unmodified pSM103 vector. The transformation efficiencies were not substantially different, ranging from 10% to 30% amongst the plates (data not shown). These results indicate the amiRNAs designed to silence the soybean *Rhg1 *locus LRR-kinase gene have a root development impact specifically in soybeans.

### No Significant Impact on SCN Resistance after Partial Silencing of the *Rhg1 *locus LRR-kinase

**Figure 6:**
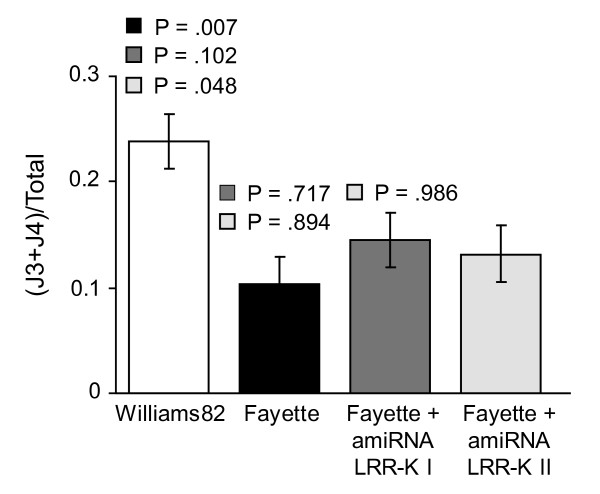
**Nematode demographic assay for SCN resistance in roots transformed with amiRNA silencing the *Rhg1*-locus LRR-kinase gene**. Ratio of total infecting nematode population that gained appearance of J3 and J4/adult female stages two weeks after inoculation onto *A. rhizogenes*-transformed roots. Williams 82 (susceptible) and Fayette (resistant) soybean tissue (left two bars) were transformed using *A. rhizogenes *carrying the pSM103 negative control plasmid; Fayette + amiRNA received the designated amiRNAs that target the *Rhg1*-locus LRR-kinase gene (amiRNA LRR-K I and amiRNA LRR-K II). Graph shows mean ± std error of mean from three independent nematode infection assays that used roots from three independent transformation experiments (overall n > 18 for each treatment). P values above each bar are color-coded for contrasts with the bar of the designated color (null hypothesis: the observed ratio between the two treatments was similar; tested by ANOVA using SAS Proc Mixed).

Healthy transgenic GFP-positive roots expressing amiRNA constructs that target the *Rhg1 *locus LRR-K gene (or empty vector controls) were sub-cultured by propagating the young growing tips (2-3 cm), and were infected with ~250 sterile J2 SCN for each root. The nematode demographics assay was then used to detect relative SCN resistance. The experiment was repeated with three independent biological replicates each using 4-10 separate transgenic hairy roots of each treatment-construct. Two weeks after nematode inoculation, all nematode stages (except eggs) were observed in the transgenic roots tested, and the number of nematodes at each stage was recorded. Roots carrying five or fewer nematodes were excluded from further analysis. As expected, Fayette control roots allowed significantly fewer nematodes to molt and progress to an apparent J3 or J4/adult female stage within 14 days relative to Williams 82 control roots (Figure 6; p = 0.007). In the key comparison to test if silencing of the *Rhg1 *locus LRR-kinase gene altered resistance to SCN, no statistical difference in nematode development was observed between Fayette and Fayette carrying amiRNA GmLRR-K I or amiRNA Gm-K II (Figure 6).

## Discussion

As one part of this study, we report use of amiRNA technology to knock down expression of an endogenous gene in soybean. Like RNAi (hairpin RNA or VIGS-mediated silencing), miRNAs and amiRNAs in plants reduce gene expression primarily by eliciting cleavage of homologous RNA transcripts. The primary benefit of adopting amiRNA technology is specificity [50-52]. Longer hairpin RNA gene silencing constructs (often 100-800 bp) are cleaved into multiple subfragments that, with the known tolerance for mismatches within the RNA-induced silencing complex, can cause unintended silencing of non-target genes. This can readily remain undetected by the investigator. The target recognition portion of miRNAs and amiRNAs are very short and are strand specific, and plant miRNA-mediated silencing is more sequence-specific than animal miRNA-mediated silencing, all of which greatly reduces the potential for unintended off-target effects on other genes. Based on known target selectivity behaviors (see for example http://wmd3.weigelworld.org/ and [50], the amiRNA can be designed to be gene specific - even targeting specific splice forms - but like other gene silencing methods, amiRNAs can alternatively be designed to silence multiple similar genes if an appropriate shared target sequence is used.

We constructed the vector pSM103 to address three goals: ready acceptance of newly generated amiRNA constructs into an *Agrobacterium *plant transformation vector, strong expression of the amiRNA in multiple tissues in soybean, and presence of an intron-disrupted GFP to allow identification of transgenic tissues. Each of these ideas has ample precedent but they had not been brought together for soybean. Gmubi is a strongly expressed promoter - offering potential improvements over CaMV 35S and other promoters for use in soybean and other legumes [54,55]. Although the Arabidopsis miRNA319a has been an effective template for successful amiRNAs used in tomato, tobacco, *Physcomitrella patens *and other species [56,57], and was effective in the present use in soybean, it is possible in the future that improved silencing with amiRNA in soybean may be achievable by using one of the many endogenous soybean miRNAs as the template [58,59]. Another permutation for future investigation may be the use of highly specific promoters to drive expression of the amiRNA, to elicit silencing in a more limited set of cells, tissues or developmental stages. However, the success of amiRNA was demonstrated in the present study by the successful reduction in target mRNA abundance and by the elicitation of root development impacts in lines carrying amiRNA LRR-K I. In current work with other soybean genes, we are finding that roughly half of the amiRNAs that we construct cause detectable reduction in target mRNA abundance (unpublished), as is commonly observed in most laboratories that work with hairpin RNAs, amiRNAs and other engineered silencing methods.

The nematode demographics assay was developed as a modification of existing protocols that monitor SCN development on transgenic soybean roots generated using *A. rhizogenes *[37,38]. There were multiple motivations for developing the demographics approach. First and most important, we hypothesized that a census of the developmental stages achieved by the infecting SCN population mid-way through the infection life-cycle might be more sensitive than an end-stage cyst count in detecting differences in the expression SCN resistance, which is a quantitative trait. Previous reports indicate that morphological differences between nematode development on SCN-resistant and SCN-susceptible soybeans are evident relatively early - as soon as 5 days after infection [38,53,60,61]. Gene expression profiling work has even suggested host gene expression differences between resistant and susceptible interactions in the first 12 hours [62]. A second motivation for the demographics assay was that root segments often became less healthy in appearance after 3-4 weeks in culture. Root tips remain vigorous and continue to grow, but under our conditions, older segments such as the segments present at the time of nematode infestation start to exhibit notable decline approximately three weeks after exposure to nematodes. Data collection at an earlier time point reduces this concern. A third useful attribute of the demographics assay is that results become available two weeks earlier, compared to cyst counts at 30 days post-infection. Lastly, when first establishing the assay in our laboratory, slow-growing fungal contaminants sometimes emerged in the third or fourth week (usually introduced by application of clean but not fully sterile nematodes). Although this issue was addressed primarily by more effective nematode surface-sterilization or use of axenic nematode cultures, use of an assay that terminates two weeks after SCN application reduced contamination issues (note that more thorough surface sterilization is lethal to a high percentage of the nematodes and introduces its own potential problems). The validity of the demographics scoring approach was supported by the significant differences in SCN development we observed between known resistant and susceptible plant genotypes. Acid fuchsin staining and nematode counting do require some additional effort, however, and previously published protocols that count cyst formation after 28-35 days remain as an acceptable alternative.

In addition to reporting amiRNA use for gene silencing in soybean, a vector to facilitate this approach, and the nematode demographics assay for tests of genes that may contribute to soybean SCN resistance, we also report negative data regarding impacts of the *Rhg1*-locus LRR-K gene on SCN resistance. Using complementation with a full-length gene under control of its native promoter, and gene knockdown using amiRNA technology, we were unable to detect a contribution to SCN resistance by the *Rhg1*-locus LRR-K gene from either Peking/PI 437654 or PI 88788, the most commonly used sources of SCN resistance in U.S. soybean cultivars. An impact on root development was observed when amiRNA for the LRR-K gene were expressed in transgenic roots, and effects on root development have also been preliminarily reported by the Lightfoot research group http://hdl.handle.net/10101/npre.2008.2726.1. It remains possible that this *Rhg1*-locus LRR-K gene may in some way impact SCN infection of or development on soybean roots, but no such effect was detected in our studies.

The *Rhg1 *locus is of extremely high economic significance to soybean cultivation in the United States, and has received attention from multiple research groups. It is notable that more than ten years after the filing of patent applications claiming SCN resistance function for the *Rhg1*-locus LRR-K gene, no groups have published evidence (beyond its presence as one of many candidate genes at the *Rhg1 *locus) for a functional contribution to SCN resistance by the *Rhg1*-locus LRR-K gene. We note that, simultaneous with the present study, a group led by Drs. Khalid Meksem and Melissa Mitchum has completed tests for functional contributions of the *Rhg4*-locus LRR-K gene to SCN resistance and, similar to our work, has not detected a contribution for this *Rhg4 *candidate gene (K. Meksem, personal communication). We are also aware of work led by Dr. Brian Diers (Kim et al., submitted)[63], in which fine-structure genetic mapping of the *Rhg1 *locus has revealed chromosomal recombination events that genetically separate *rhg1-b *(PI 88788-source) SCN resistance function from the *Rhg1*-locus LRR-K gene. Hence evidence is accumulating that the *Rhg1 *locus LRR-K genes seem unlikely to be the primary source of SCN resistance polymorphism. A tone of caution is appropriate, however, as our present study only provides negative data on this subject. For example, gene silencing is often partial (whether mediated by VIGS, hairpin RNA-generating constucts or amiRNA), and the expression knockdown we achieved with amiRNA may have been insufficient to detectably block hypothesized SCN resistance contributions (although the other phenotypic impacts we observed with the amiRNA LRR-K I suggest that the gene expression knockdown achieved was functionally significant). As another example, the quantitative nature of the functional contribution of any given gene to SCN resistance may be difficult to detect in *A. rhizogenes*-generated soybean roots. Hence we are cautious with our conclusion, but we can state that when SCN-susceptible sources were complemented with an *Rhg1*-locus LRR-K allele from an SCN-resistant source, or when expression of the gene in resistant plants was reduced by amiRNA-mediated silencing, no significant impacts on SCN resistance were detected.

## Conclusions

Our findings suggest that the nematode demographics assay can expedite testing of transgenic roots for SCN resistance. amiRNAs may have widespread use in legume biology, and we have developed the vector pSM103 that drives interchangeable amiRNA constructs through a Gmubi promoter, with an intron-GFP marker for detection of transgenic roots. Studies in which expression of the *Rhg1 *locus LRR-kinase gene from different resistance sources was either reduced or complemented did not reveal significant impacts on SCN resistance, suggesting that one or more other genes at the *Rhg1 *locus may be the primary determinant of differences in SCN resistance among different soybean types.

## Methods

### *Agrobacterium *cultures

*A. tumefaciens GV3101 *and *A. rhizogenes *strain Arqua1 were transformed by freeze-thaw [64] or by electroporation [65]. The cells were plated on selective media with the appropriate antibiotic and incubated at 28°C for about 2 days. *A. rhizogenes *strain Arqua1 was received from Dr. Jean-Michel Ane, University of Wisconsin Madison.

### Soybean lines

Soybean cultivars Fayette and Ina (SCN-resistant lines), and Williams 82 and E98076 (susceptible lines) were used; E98076 is an SCN-susceptible experimental line from Dechun Wang, Michigan State University. Genotyped Ina × E98076 progeny lines were kindly provided by Dr. Brian Diers, University of Illinois at Urbana-Champaign. Those lines were inferred to be homozygous for *Rhg1*_Ina _or *rhg1*_E98076_, and for *Rhg4*_Ina_, or *rhg4*_E98076_, using micro satellite markers Satt309 and Satt424 [66]; http://soybeanbreederstoolbox.org/. DNA sequencing was used to confirm nucleotide identity between the *Rhg1*-locus LRR-kinase gene from cultivars Ina and Peking.

### Surface sterilization

After visual screening for obvious signs of fungal or viral contamination, soybean seeds were surface-sterilized for 16-20 h in a desiccator jar with chlorine gas by adding, to a beaker within the jar, 3.5 ml 12 N HCl into 100 ml household bleach (6% sodium hypochlorite) immediately before closing the jar. At least 20 seeds per experiment were plated onto germination media (Gamborg's B5 salts (3.1 g/L), 2% sucrose, 1 × Gamborg's B5 vitamins, 7% Noble agar, pH 5.8) in 100 × 25 mm Petri plates. Plates were wrapped with Micropore tape (3M, St. Paul, MN) and incubated at 26°C in a growth chamber (18/6 light/dark hours) for about a week. *Medicago truncatula *seeds (approximately 150 per batch) were scarified in concentrated sulfuric acid for 8 min, washed 3 times in sterile water, plated on 1% deionized water agar plates, and then stratified for 24 h or 48 h at 4°C and germinated by incubating at room temperature overnight [67].

### Soybean and *Medicago *transformation

Soybean cotyledons were harvested about 7 days after germination by gently twisting them off the hypocotyls. With a sterile forceps and scalpel, several shallow slices were made across the abaxial surface of the cotyledons after dipping the scalpel in *A. rhizogenes *suspension (OD600 0.7 in Luria broth). The cotyledons were then placed abaxial-side down on a co-culture medium (0.31 g/L Gamborg's B5 salts, 3% sucrose, 1 × Gamborg's B5 vitamins, 0.4 g/L L-cysteine, 0.154 g/L dithiothreitol, 0.245 g/L sodium thiosulfate, 40 mg/L acetosyringone, 5% Noble agar, pH 5.4) in 100 × 15 mm Petri plates with a piece of 70 mm filter paper (Whatman, Piscataway, NJ) on the surface of the agar to prevent *A. rhizogenes *from overgrowing. Plates were wrapped with parafilm and incubated in the dark at room temperature for three days. The explants were then transferred to a hairy root medium (HRM) of 4.3 g/L MS salts (Sigma Co., St. Louis, MO), 3% sucrose, 1 × Gamborg's B5 vitamins (Sigma Co. St. Louis, MO), 7% Noble agar, 0.15 g/L cefotaxime, 0.15 g/L carbenicillin, pH 5.6 in 100 × 15 mm Petri plates, wounded side up. Plates were wrapped with Micropore tape and incubated in the dark at room temperature until roots emerged, usually in around 3 weeks.

To generate *M. truncatula *with transgenic roots, *A. rhizogenes *Arqua1 carrying pSM103 with one of the two different amiRNA for the *Rhg1 *GmLRR-Kinase (amiRNA GmLRR-KI and GmLRR-KII), or the empty vector, were used for *M. truncatula *transformation [68]. Following transformation, seedlings were grown for 3 weeks. The bottom part of the plates (corresponding to the roots) was covered with aluminum foil. The plates and the *M. trucatula *composite plants were watered with sterile water every other day.

### Transgenic root identification and propagation

Soybean or *M. truncatula *transgenic roots were detected based on GFP expression, using a fluorescence stereomicroscope (LEICA MZ FL III with GFP2 filter). Transgenic soybean root tips were cut into 2-3 cm segments and transferred to HRM. Roots that were expressing incomplete strips of fluorescence (chimeras) or exhibiting overall low levels of GFP fluorescence were avoided. GFP-positive as well as negative control roots were generally used within two months of the initial transformation event, after maintenance by transferring 2-3 cm root tips every two weeks. Independent transgenic events, generated from different inoculation sites or different cotyledons, were maintained separately for use in RT-PCR and nematode demographic assays.

### Cloning of candidate *Rhg1 *LRR-kinase gene

The LRR-kinase gene Glyma18g02680.1 from the *Rhg1 *locus was cloned from Ina genomic DNA. Primers were designed based on BAC sequences from soybean cultivar A3244 [30], using the Primer3 tool. The Roche Expand Long™ (Roche Applied Science, Indianapolis, IN) kit and protocol were used with primers

Rhg1LRR-KF: TTCACCCGTGATACATGTTAATTC;

Rhg1LRR-KR: GGAACTTGGAAGTCATTATCTTGG

to amplify a 6.9 kb PCR product from the *Rhg1 *locus that includes >2 kb of upstream sequence, the coding region, and 0.8 kb of downstream sequence, which was then cloned into the Invitrogen plasmid pCR8/GW/TOPO according to the manufacturer's instructions. Multiple independent clones were isolated and sequenced in order to avoid clones with PCR-induced mutations. The binary vector pGWB1::erGFP7INT for full-length gene complementation experiments was constructed from pGWB1 [69] by addition of the 35S-promoter/erGFP7INT/NOS-terminator of pLMNC95 [70], taken from a pCAMBIA 1300 series vector by digestion with EcoRI and HindIII and inserted into the HindIII site of pGWB1 after blunting. The LRR-kinase genes (in pCR8/GW/TOPO) from the *Rhg1 *locus were moved in to pGWB1::erGFP7INT using the Invitrogen Gateway™ system (Invitrogen, Carlsbad, CA) after linearizing the pCR8/GW/TOPO-derived plasmids with XbaI. To obtain a negative control transformation vector lacking the Gateway *ccdB *negative selectable marker, a 167 bp segment of nonsense DNA was cloned and transferred into pGWB1::erGFP7INT.

### Nematode maintenance

A Wisconsin SCN population (Hg type 7), collected by A.E.M., was maintained on the susceptible soybean cultivar Williams 82. Seeds were germinated in a wet 70 mm filter paper (Whatman) in a Petri plates for 4-5 days, planted in autoclaved 2:1 sand:soil mixture, inoculated with 2000-3000 eggs of *H. glycines *per container, and grown in a water bath at 28°C in a 28°C greenhouse. Cysts were collected 6 weeks after infection, and were extracted from infested pots using sieves and centrifugation. Briefly, soil from infected pots was placed in a pitcher of water and the roots were massaged to dislodge attached cyst. The soil-cyst-water slurry was passed over a 595 μm - 250 μm sieve tower, and the mixture from the 250 μm sieve was backwashed into a 50 mL plastic conical tube. The tubes were centrifuged at 2000 RPM for 4 minutes then the supernatant was poured off. A 60% sucrose solution was added to the tubes, stirred, and centrifuged at 2000 rpm for 2 min. Cysts in the supernatant were then collected over a 250 μm sieve. Collected cysts were stored at 4°C in plastic bags in damp sand.

### Nematode demographics assay

For nematode demographics assays, *H. glycines *eggs were collected by breaking open cysts and collecting the eggs on a sieve stack consisting of 250 μm - 74 μm - 25 μm sieves (USA Standard Testing Sieve). Eggs were collected from the 25 μm sieve and rinsed. Eggs were placed in a hatch chamber [71] with 3 mM ZnCl_2 _for hatching at room temperature in the dark for 5-6 days. Hatched J2 nematodes were surface-sterilized for 3 min in 0.001% mercuric chloride and washed three times with sterile distilled water [72], then suspended in 0.5% low-melting point agarose to facilitate even distribution. The number of active nematodes was counted using a hemacytometer at least one-half hour after surface-sterilization and washing, and 250-500 active J2s were inoculated onto each fresh root segments, depending on the experiment (similar numbers of active J2 were applied to all roots within an experiment). Roots (2-3 cm vigorous new root segments closest to and including root tip) with nematodes were maintained on HRM media at 28°C (in initial experiments including some experiments reported in Figure 1, roots were placed on cellophane on HRM media for 24 hr. starting just before nematodes were added, and then transferred to HRM without cellophane and incubated vertically for 48 hr., then incubated horizontally until experiment was complete). Nematode infection and development within root segments was monitored by clearing and staining with acid fuchsin as in [73], typically 15 days post inoculation (dpi). The nematode demographic assay was then completed by recording the number of nematodes in each root with a morphology resembling either J2 (thin), J3 (sausage-shaped) or J4/adult female nematodes, as noted in text and figures.

### Construction of amiRNA vector

To construct the pSM103 vector for amiRNA-mediated gene silencing in legume roots (Figure 2), the 35S-promoter/erGFP7INT/NOS-terminator construct (intron-GFP) from pLMNC95 [70] was mutagenized to remove XbaI and BamHI sites, and then cloned from pLMNC95 (ΔXbaI and ΔBamHI) into pCAMBIA 1300 after PCR-mediated addition of restrictions sites using the primers:

Plmnc95_Kpn1F: CCAGGTACCCAGGTCCCCAGATTAGCC;

Plmnc95EcoR1_R: GCCAGTGAATTCCCGATCTA.

The amiRNA constructs from pRS300 derivatives (see below) were added to pCAMBIA 1300::35SP-erGFP7INT-NOS using BamHI/PstI sites. The soybean polyubiquitin (gb|EU310508.1|) promoter (GmUbi) [54,55] was amplified from Fayette genomic DNA with added HindIII and PstI sites using the primers:

Ubi1_HindIII_Fa: CCAAAGCTTGGGCCCAATATAACAACGAC;

Ubi_PstI_Ra: CCACTGCAGCTGTCGAGTCAACAATCA.

The Gmubi product was cloned into pCR2.1 TOPO and then in pCAMBIA 1300::35SP-erGFP7INT-NOS::miR319a using HindIII/PstI sites.

### Transient Expression Assay

The plasmids pGWB1::erGFP7INT, pSM101 and pSM103 were initially tested for GFP function by agroinfiltration of *A. tumefaciens *GV3101 into *Nicotiana benthamiana *leaves, along with GV3101 without binary plasmid as a negative control. *A. tumefaciens GV3101 *strains carrying pGWB1::erGFP7INT, pSM101 and pSM103 were grown overnight at 28°C in 5 ml LB medium with 2.5 μg/ml of rifampicin and 50 μg/ml of kanamycin (the negative control *A. tumefaciens *GV3101 without binary vector was grown only with rifampicin). *A. tumefaciens *cells were harvested by centrifugation and resuspended to OD_600 _0.4 in MMA induction buffer (5 g MS salts, 20 g sucrose, 1.95 g MES per liter, 200 μM acetosyringone, pH to 5.6 with 1M NaOH. Bacterial suspensions were then incubated at room temperature for 1-3 hr prior to infiltration through the abaxial surface into the leaf, using a syringe with no needle. GFP fluorescence was investigated 3 days after infiltration in excised leaf sections that included the infiltrated zone and 1-3 cm of surrounding non-infiltrated tissue, using an Olympus BX-60 upright Fluorescence Microscope and MF filter.

### Sequencing and amiRNA design

EST TC204550 UP|Q8L3Y5 (Q8L3Y5) (Glycine max EST GmGI-12.0; wmd2 database http://wmd2.weigelworld.org/cgi-bin/mirnatools.pl) corresponding to the Glyma18g02680.1 putative LRR-kinase gene from the *Rhg1 *locus was used to design PCR primers for isolation of the *Rhg1 *locus receptor-like kinase-encoding allele from the resistant source Fayette, which carries the *Rhg1 *resistance allele *rhg1-b *from PI 88788. The primers RTPCR_TC204550F CAA CTT CAA GGC CTC AGG AA; RTPCR_TC204550R GCT ACC CAA AGA AGC AGG AA, amplified a PCR product of 450 bp. The DNA sequences of PCR products were determined by the dideoxy chain-termination method using ABI big dye cycle sequencing kit (3.1) and the ABI 377 automated sequencing service at the University of Wisconsin - Madison Biotechnology Center. Blast analysis of the Fayette version of TC204550 (the EST for this gene in earlier WMD versions) was conducted in March 2008 using the Phytozome database http://www.phytozome.net/soybean. For amiRNA design, the sequence of this Fayette LRR-kinase gene was submitted to http://wmd2.weigelworld.org. The wmd2 tool identified a total of 85 candidate amiRNA sequences to potentially silence the *Rhg1 *locus LRR-kinase gene, with the first 31 candidates classified by wmd2 as having excellent (most promising) properties (data not shown). The two amiRNA target sequences selected for primer design to modify pRS300 for silencing of the *Rhg1 *locus LRR-kinase gene were identical to Fayette chromosome 18 sequence as well as the paralogous chromosome 11 sequence from the Williams 82 genome: amiRNA GmLRR-KI: TAAGACTATAAGGGATTGCTG; and amiRNA LRR-KII: TAAGACTATAAGGGATTGCTC. The Arabidopsis miR319a sequence from pRS300 was used as a template to create three PCR products (a, b, c) with soybean target transcript specificity. These products were used as template for a fourth PCR using overlapping primers to create gene specific amiRNA [51]. The final PCR product (containing target transcript sequence) was cloned into pSM103 using BamHI-PstI restriction sites, replacing the miR319a sequence. Presence of the desired amiRNA was confirmed by DNA sequencing.

### DNA and RNA extraction and RT-PCR

Soybean genomic DNA was extracted from young leaves using a CTAB method previously described [74]. Total RNA was extracted from independent transgenic hairy roots (about 3 cm long) that had been frozen in liquid nitrogen and stored at -80°C until use. Total RNA was isolated using Qiagen RNeasy Mini Kit according to the manufacturer's instructions, and treated with RNase-free DNase I (Qiagen, Valencia, CA, U.S.A.). First-strand cDNA was synthesized from 250 ng of DNase-treated RNA using SuperScript III Reverse Transcriptase. The RNA concentration was determined using the NanoDrop-1000 Spectophotometer. Semi-quantitative RT-PCR was performed using 25 ng of cDNA. Primers designed from the Fayette *Rhg1 *locus LRR-Kinase target, RTPCR_TC204550F CAACTTCAAGGCCTCAGGAA (forward) and RTPCR_TC204550R GCTACCCAAAGAAGCAGGAA (reverse), were used to amplify a 450 bp product. As a control for equivalent starting pools of cDNA, the primers Actin forward GTTCTCTCCTTGTATGCAAGTG and Actin reverse CCAGACTCATCATATTCACCTTTAG were used to amplify a 700 bp segment of soybean actin gene cDNA (emb|V00450.1|) The RT-PCR was performed in triplicate at 55°C, testing three different numbers of cycles (30-35-40) to confirm non-saturation of amplification. PCR products were visualized by ethidium bromide staining after separation on 1.5% agarose gels and band intensity was quantified using the Kodak 1D Image Analysis Software v3.6.

### Statistical analysis

Data were analyzed by ANOVA using Minitab (v.14) with the General Linear Model and Tukey Simultaneous Test; nematode demographics data for the amiRNA experiments were analyzed using a mixed model (Mixed Procedure; SAS, vers. 9.1).

## Authors' contributions

SM, ALH and DC planned, conducted and analyzed most of the experiments and were centrally involved in writing the manuscript. BWD developed soybean germplasm and provided significant ideas and critical review of the manuscript. AEM contributed nematode populations, methodological suggestions and biological insights. AFB conceived the overall project, analyzed results and planned experiments, and was a primary author of the manuscript. All authors read and approved the final manuscript.
